# Comparison of dyad versus individual simulation-based training on stress, anxiety, cognitive load, and performance: a randomized controlled trial

**DOI:** 10.1186/s12909-021-02786-6

**Published:** 2021-07-05

**Authors:** Eduardo F. Abbott, Torrey A. Laack, Lauren K. Licatino, Christina M. Wood-Wentz, Paul A. Warner, Laurence C. Torsher, James S. Newman, Katie M. Rieck

**Affiliations:** 1 Multidisciplinary Simulation Center, Mayo Clinic, 200 First Street SW, Rochester, MN 55905 USA; 2 Department of Internal Medicine, Escuela de Medicina, Pontificia Universidad Catolica de Chile, Diagonal Paraguay 362, 5th Floor, 8330077 Santiago, Chile; 3 Department of Emergency Medicine, Mayo Clinic, Rochester, MN USA; 4 Department of Anesthesia and Perioperative Medicine, 200 First Street SW, Rochester, MN 55905 USA; 5 Division of Clinical Trials and Biostatistics, Mayo Clinic, 200 First Street SW, Rochester, MN 55905 USA; 6 Division of Hospital Internal Medicine, Mayo Clinic, 200 First Street SW, Rochester, MN 55905 USA

**Keywords:** Anxiety, Cognitive load, Dyad, Simulation, Stress

## Abstract

**Background:**

Dyad learning has been shown to be an effective tool for teaching procedural skills, but little is known about how dyad learning may impact the stress, anxiety, and cognitive load that a student experiences when learning in this manner. In this pilot study, we investigate the relationship between dyad training on stress, anxiety, cognitive load, and performance in a simulated bradycardia scenario.

**Methods:**

Forty-one fourth-year medical school trainees were randomized as dyads (*n* = 24) or individuals (*n* = 17) for an education session on day 1. Reassessment occurred on day 4 and was completed as individuals for all trainees. Primary outcomes were cognitive load (Paas scale), stress (Cognitive Appraisal Ratio), and anxiety levels (abbreviated State-Trait Anxiety Inventory). Secondary outcomes were time-based performance metrics.

**Results:**

On day 1 we observed significant differences for change in anxiety and stress measured before and after the training scenario between groups. Individuals compared to dyads had larger mean increases in anxiety, (19.6 versus 7.6 on 80-point scale, *p* = 0.02) and stress ratio (1.8 versus 0.9, *p* = 0.045). On the day 4 post-intervention assessment, no significant differences were observed between groups. Secondary outcomes were significant for shorter time to diagnosis of bradycardia (*p* = 0.01) and time to initiation of pacing (*p* = 0.04) in the dyad group on day 1. On day 4, only time to recognizing the indication for pacing was significantly shorter for individual training (hazard ratio [HR] = 2.26, *p* = 0.02).

**Conclusions:**

Dyad training results in lower stress and anxiety levels with similar performance compared to individual training.

**Supplementary Information:**

The online version contains supplementary material available at 10.1186/s12909-021-02786-6.

## Background

Collaborative learning, understood as multiple learners engaged in an educational endeavor collectively, is a common strategy in education. In health professions education (HPE), this methodology is commonly used for problem-based learning and team-based learning [1–3]. There is a nascent body of evidence in HPE that clinical and procedural skills can also be developed via collaborative learning. Most of this emerging, collaborative learning literature examines simulation based medical education (SBME) of dyads (paired collaborative learners) versus individuals, with results demonstrating higher learner satisfaction, an equivalent educational effect, and more efficient and cost-effective training for dyads [4–8]. Proposed mechanisms of dyad learning include social interaction, positive interdependence among trainees, observational learning (action imitation), and shared knowledge [9, 10]. These potential benefits come at the cost of reduced individual “hands-on” time, which may lessen the benefits of dyad learning [9]. In addition, concern exists that some learners will be less engaged and depend heavily on their partner, ultimately reducing the efficacy of the learning experience. The optimum type of content and means for implementing dyad learning has not yet been clarified; the majority of published studies are on procedural topics, where motor learning is the primary objective.

Of the numerous elements that affect learning, emotional state and cognitive load have not been thoroughly addressed in the dyad learning literature for HPE. The ideal types and amounts of stressors to optimize retention of medical education also have not been determined. Attention to these potential mediators of the learning process has resulted in, at times, conflicting results in the HPE literature regarding whether stress and anxiety have positive or negative effects on learning [11, 12]. While there are inconsistent results in the literature, studies show that during high stress situations, performance declines on tasks that require divided attention. Working memory and memory recall can also be negatively affected, though some studies showed that memory consolidation (the process of creating stable memories for future retrieval) may be enhanced [11, 13–15]. LeBlanc notes memory consolidation occurs best at moderate stress levels; extremely high stress levels will impair this process. In addition, it is important that the source of stress relate directly to the clinical case itself rather than the stress of being observed by a supervisor or teacher, as the latter would result in the encoding of memory related to the stress of the observation rather than knowledge that came from working through a challenging patient scenario [11].

Cognitive load theory states that human cognitive processing system has finite capacity and theorists refer to any burden placed upon this system as ‘cognitive load’ [16]. During learning, some cognitive load is inherent to the material being learned (intrinsic load), some is related to the effort of learning (germane load), and some is caused by extraneous factors (extrinsic load) [17]. Since excess cognitive load may impair learning, reduction in overall cognitive load is a desirable outcome [18–20]. On the other hand, practicing and learning under conditions of increased cognitive load – especially those that are similar to real life clinical encounters—may allow practice and adaption to these challenging settings.

To our knowledge, the use of dyad training has not been compared to individual training for measurement of trainees’ stress, anxiety, or cognitive load on a complex task. Dyad training has also not been extensively studied in non-procedural clinical skills. We hypothesized that during the initial learning session, medical students who complete dyad bradycardia simulation-based scenario training will have lower stress, anxiety, and cognitive load levels compared to students undergoing individual training, and that dyads would perform better when compared directly to individuals. Because some of those who initially trained in pairs may have relied on their partners and not been as engaged in the learning process, we also hypothesized that on post-intervention assessment as individuals 3 days later, those who trained as dyads would demonstrate a decrease in performance metrics (including time to diagnosis, time to pacing, and an objective checklist on transcutaneous pacemaker placement) compared to those who participated initially as individuals.

## Methods

This study is a randomized prospective trial to compare the effects of dyad versus individual learning during a simulation-based bradycardia scenario on emotional state (stress, anxiety), cognitive load, and educational benefit (performance). The study was deemed to be exempt from approval by our institutional review board (IRB).

### Participants and setting

Study participants were fourth-year medical students from the Mayo Clinic School of Medicine, in the final months of medical school training. All fourth-year students take part in a mandatory annual one-week experience known as “Internship Boot Camp” (IBC) which has previously been described [21]. To maximize experiential learning, the course is run in sessions that range from 7 to a maximum of 16 students per week. IBC is set up as a simulated inpatient medical service, where students are called upon to manage dozens of common inpatient issues for patients on their service, including chest pain, hyperkalemia, and post-operative pain. Although the educational sessions were mandatory, students were made aware that their participation in the research study was not required and would not impact their grade. All students voluntarily chose to participate in the research surveys. All Mayo Clinic School of Medicine students have numerous required educational activities at the Simulation Center and are familiar with this educational environment.

All students (*n* = 41) were randomly assigned to undergo a simulation-based bradycardia scenario (day 1) as either an individual (*n* = 17) or dyad (*n* = 24). Sample size was limited to 41 by the size of the 2017 graduating class. No formal power calculation was done due to the limitations of the sample size in this exploratory study. The entire class participated. A randomization schedule was prepared by members of the Division of Clinical Trials and Biostatistics using SAS software (SAS Institute Inc., Cary, NC). In order to accommodate the possibility that responses for pairs of students who work in dyads would be correlated, the total number of individuals randomized to dyads was increased under the assumption that the intracluster correlation coefficient ICC was approximately 0.33. Based on this assumption, a total of 24 individuals assigned to dyads would result in an effect sample size of *N* = 18 for this group.

Given the large size of most medical school classes (relative to residency or fellowship classes), and limited financial and time resources within such programs, we felt that medical students would be an ideal study population for assessment of dyad training. The symptomatic bradycardia scenario was chosen because management of bradycardia is one of the required skills of a proficient Advanced Cardiac Life Support (ACLS) provider. Most interns will be expected to complete ACLS training either prior to starting or early on in their internship. Bradycardia presents a high intensity clinical scenario that is challenging and is a valuable educational addition for all of our senior medical students that was not previously part of the curriculum. In addition, the naturally required interactions between the medical student learners and actors (nurse and deteriorating patient with bradycardia) necessitates interdependence between the learners in the dyad group, including acquiring, setting up, and operating the pacing device.

### Study materials and procedure

Baseline stress and anxiety assessments (Cognitive Appraisal Ratio [CAR] and abbreviated State-Trait Anxiety Inventory [STAI], respectively) were performed prior to the initial scenario. Students were then given a brief written summary of the simulated patient’s medical history and current vitals; dyads reviewed this together. Students were then called into the simulation room to evaluate the patient who was not feeling well. The patient had third-degree atrioventricular block (i.e., complete heart block) with a heart rate of 30 displayed on the monitor. The goal of the scenario was for the students to recognize that the patient is having symptomatic bradycardia and to employ transcutaneous pacing after an appropriately focused history and examination. A SimMan 3G advanced patient simulator (Laerdal Medical®) was used for all sessions. The same script was used for the simulated patient responses voiced by one of two investigators (TAL or EFA) in all scenarios. See Additional file 1: Appendix I for script.

**Fig. 1:**
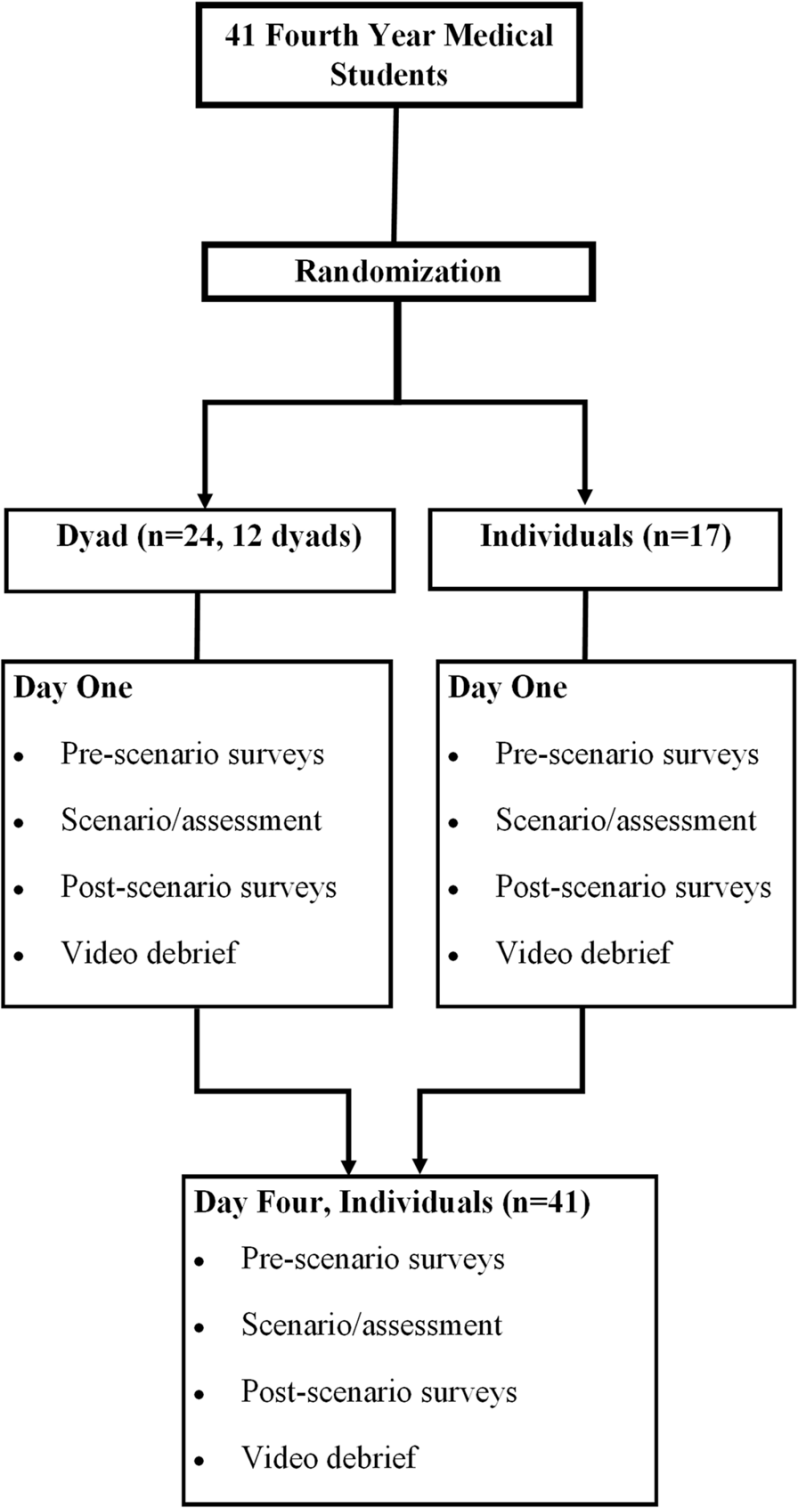
Study Timeline

Immediately following the training scenario and before leaving the simulation room, assessments of stress (CAR), anxiety (STAI), and cognitive load (Paas) were collected. All survey scales were completed individually regardless of the study group assigned. All students then watched an approximately 8-min debrief video with no individualized debriefing or feedback (see Additional file 1: Appendix II). A video was selected to avoid confounding related to students receiving differing debriefing sessions. Three days later (day 4), all learners experienced a nearly identical scenario as individuals (only the patient name, age, and comorbidities were changed) as a post-intervention assessment. Day 4 method of survey assessment prior to and following the scenario was nearly identical to day 1, with the only exception being that trainees were also surveyed regarding what type of training they thought they preferred (individual vs dyad training). See Additional file 1: Appendix III for survey instruments. Students had no prior knowledge of the content or topic for the sessions on day 1 or day 4. Following the simulation assessment of symptomatic bradycardia management and data collection on day 4, a course instructor (TAL or KMR) provided individualized debriefing and feedback to each student to consolidate learning and provide a formative structure. The timeline in Fig. 1 summarizes the study flow.

All learners were assessed on management of symptomatic bradycardia with a standardized transcutaneous pacemaker checklist, along with time performance metrics on time to diagnosis, time to initiating pharmacological treatment (if selected), time to knowing an indication to pace was present, and time to effective transcutaneous pacing (if selected). Time metrics and pacing effectiveness were scored by one of five investigators (EFA, KMR, TAL, JSN, LKL). Scoring was done during the live session, with video review only if scoring documentation was unclear or missing. See Additional file 1: Appendix I for checklist/scoring tool (imbedded within scenario script). Secondary outcomes were measured during the task using a stopwatch and completing the transcutaneous pacing checklist developed by Ahn [22] which was slightly adapted for our use (removing the requirement for informed consent, procedural pause and documentation); the checklist items were assessed with a yes/no score. See Additional file 1: Appendix I for scoring tool.

### Outcome measures

#### Primary outcomes

Primary outcomes for this study were cognitive load, stress level, and anxiety level. Cognitive load was measured with the Paas cognitive load scale. Cognitive load cannot be directly measured, so indirect measurements have been developed that include subjective rating scales, physiological indices, and secondary task performance; the cognitive load scale developed by Paas [23] has been commonly used in HPE literature. The Paas cognitive load scale is based on a 9-point scale, where higher scores mean increased cognitive load.

Subjective rating scales such as the Cognitive Appraisal Ratio (CAR) developed by Tomaka et al. [24, 25] are among the most commonly used and best validated in HPE literature to assess stress [26–28], and there has been an association of this tool to expected physiological reactions to stress (e.g., altered heart rate and cardiac output) [29, 30]. Stress was estimated with the CAR calculating the ratio of perceived demands to perceived resources for each student on a 6-point scale for each question, where a ratio above 1 is suggestive of stress.

Anxiety was assessed with the abbreviated 6-item STAI [31] (α = 0.82), which has been increasingly used in medical education [32–34] and utilizes less time to complete than the 20-item State Trait Anxiety Index (STAI) [35], which is a widely used assessment tool of subjective anxiety responses in HPE and other research fields.

#### Secondary outcomes

Secondary Outcomes included multiple time metrics, including: amount of time it took student(s) to diagnose bradycardia, time to recognition of need for transcutaneous pacing (TP), time to effective TP, and time to call a rapid response team (RRT) or code team. Additional secondary outcomes were student reporting of their preferred training type (individual vs dyad).

#### Other measures

Other measures collected via College of Medicine databases were student gender and their chosen residency specialty according to recent residency match information.

### Statistical analysis

Pre/post training survey responses were described as mean ± standard deviation. Comparisons for stress, anxiety, and cognitive load measures on day 1 and day 4 between individuals and dyads were done using equal variance two sample t-tests. For secondary outcomes, time to event analyses were completed utilizing Cox proportional hazards models, univariate within a session, and three parameter interaction models (day, treatment, day*treatment). The multiple observations per person were accounted for with a robust sandwich estimator for the variance estimates. Cox models were to utilize the information inherent in the instances of participants being unable to finish their task within the maximum allotted time (e.g., to be censored), while the robust sandwich estimator adjusted for the artificially deflated variability of observations within dyads and across days. The time to event univariate models within a session were visualized using Kaplan-Meier curves. Preference for training type was assessed between group assignments with a chi-square test. Statistical tests were all 2-sided, and *p*-values less than 0.05 were considered statistically significant. All analyses were done using SAS v9.4 (SAS Institute Inc., Cary, NC).

## Results

Baseline characteristics including gender as well as stress and anxiety levels assessed prior to starting the first session on day 1 were similar among both groups (Table 1).

**Table 1:** Baseline Characteristics

	Individual (*N* = 17)	Dyad (N = 24)	*p* value
**Males**	10 (59%)	12 (50%)	
**Residency Match plans**			
Psychiatry	1	4	
Neurosurgery	1	2	
Dermatology	2	2	
General surgery	2	1	
Pediatrics	3	2	
Radiology/Rad Onc	2	1	
Family medicine	0	2	
Internal medicine	0	4	
Orthopedic Surgery	2	0	
Urology	0	1	
Ophthalmology	1	0	
Phys Medicine &Rehab	1	0	
Plastic surgery	0	1	
OB-Gynecology	0	2	
Anesthesia	0	2	
Undifferentiated	2	0	
**Anxiety score**^a^**- Pre Day 1** Mean (SD)	39.2 (10.4)	44.6 (12.8)	0.16^1^
**Stress level ratio**^b^**- Pre Day 1** Mean (SD)	1.1 (0.5)	1.2 (0.7)	0.69^1^

*SD =* Standard deviation

^a^Anxiety score = abbreviated State-Trait Anxiety Inventory (STAI) survey

^b^Stress level ratio = Cognitive Appraisal Ratio (CAR) survey

### Primary outcomes

On day 1, we observed a significant change in anxiety and stress measures pre and post simulation session in both groups. The individuals had a larger mean increase in anxiety following the session: 19.6 ± 15.8 versus 7.6 ± 14.4 for the dyads on an 80-point scale; the differences between groups was statistically significant (*p* = 0.02). Similarly, trainees in the individual group compared to the dyad group had a larger mean increase in stress: CAR 1.8 ± 1.8 versus 0.9 ± 1.2; the difference in this ratio among groups was also statistically significant (*p* = 0.05). Cognitive load was assessed after each session and was similar between groups (6.8 ± 1.8 for individuals vs 6.8 ± 1.5 for dyads, *p* = 0.89). Complete survey results are available in Table 2.

**Table 2:** Day 1 and Day 4 Survey Results

	Individual (***n*** = 17)	Dyad (***n*** = 24)	***p*** value
**Anxiety score-Pre Day 1-**Mean (SD)	39.2 (10.4)	44.6 (12.8)	0.16^1^
**Stress ratio –Pre Day 1-**Mean (SD)	1.1 (0.5)	1.2 (0.7)	0.69^1^
**Anxiety score-Post Day 1-**Mean (SD)	58.8 (17.1)	52.2 (12.8)	0.17^1^
**Stress ratio- Post Day 1-** Mean (SD)	3.0 (2.0)	2.1 (1.3)	0.10^1^
**Cognitive load – Day 1-** Mean (SD)	6.8 (1.8)	6.8 (1.5)	0.89^1^
**Anxiety score- Pre Day 4-** Mean (SD)	39.4 (10.3)	40.0 (12.9)	0.88^1^
**Stress ratio – Pre Day 4-** Mean (SD)	1.2 (0.5)	1.1 (0.5)	0.54^1^
**Anxiety score- Post Day 4-**Mean (SD)	43.1 (12.2)	45.6 (14.9)	0.58^1^
**Stress ratio- Post Day 4-**Mean (SD)	1.1 (0.6)	1.3 (0.9)	0.42^1^
**Cognitive load – Day 4-**Mean (SD)	5.7 (2.1)	6.3 (1.4)	0.28^1^
**Preferred training**			
Response: Individual	13 (76.5%)	10 (41.7%)	**0.03**^**2**^
Response: Dyad	4 (23.5%)	14 (58.3%)	

^1^Equal Variance T-Test

^2^ Chi-Square

On day 4, we observed a smaller increase in mean anxiety following the session: 3.7 ± 17.8 for individuals versus 5.6 ± 18.8 for dyads, and minimal changes in stress for both groups, CAR -0.1 ± 1.0 versus 0.2 ± 1.0 for individuals and dyads respectively. The differences between the study groups for both anxiety and stress were not statistically significant (*p* = 0.76 and 0.37, respectively). Cognitive load continued to be similar between individuals and dyads after day 4’s session (5.7 ± 2.1 vs 6.3 ± 1.4, respectively, *p* = 0.28). Change in cognitive load between day 4 and day 1 was also not statistically significant (*p* = 0.39).

### Secondary outcomes

**Fig. 2:**
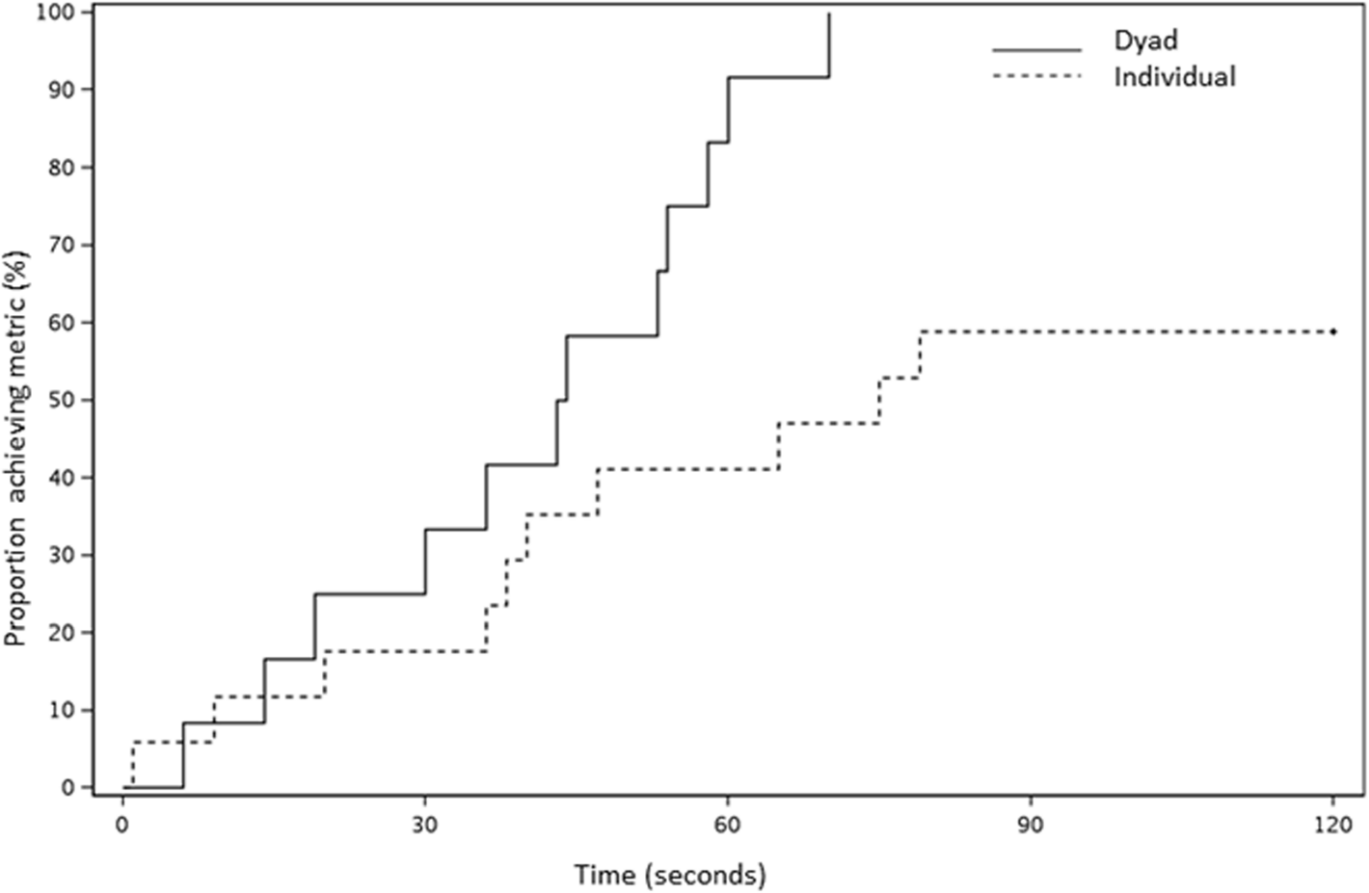
Kaplan-Meier curve of time (in seconds) to diagnosis of bradycardia, Session #1, Dyads vs Individuals

**Fig. 3:**
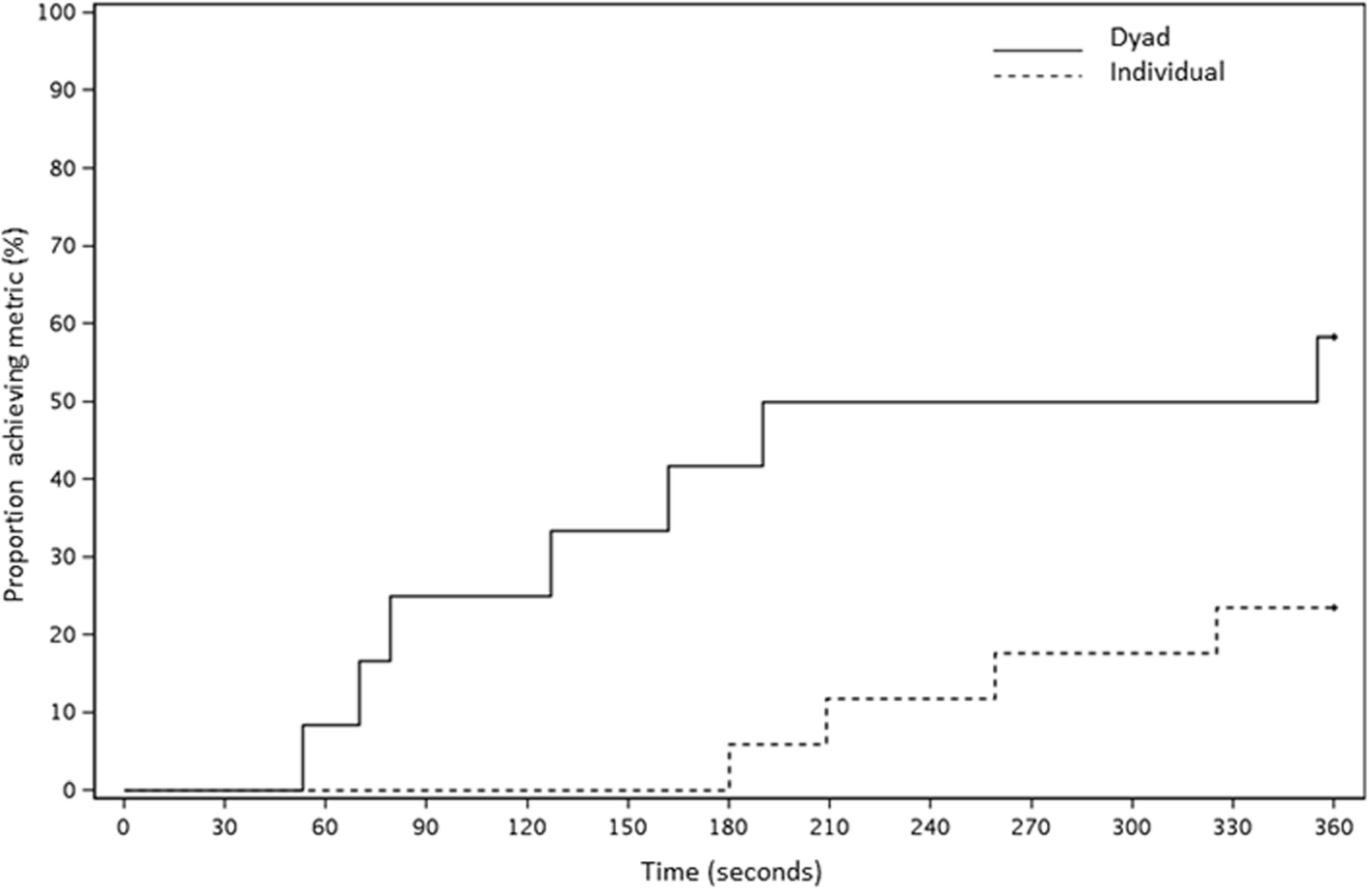
Kaplan-Meier curve of time (in seconds) to recognizing indication for transcutaneous pacing, Session #1, Dyads vs Individuals

**Fig. 4:**
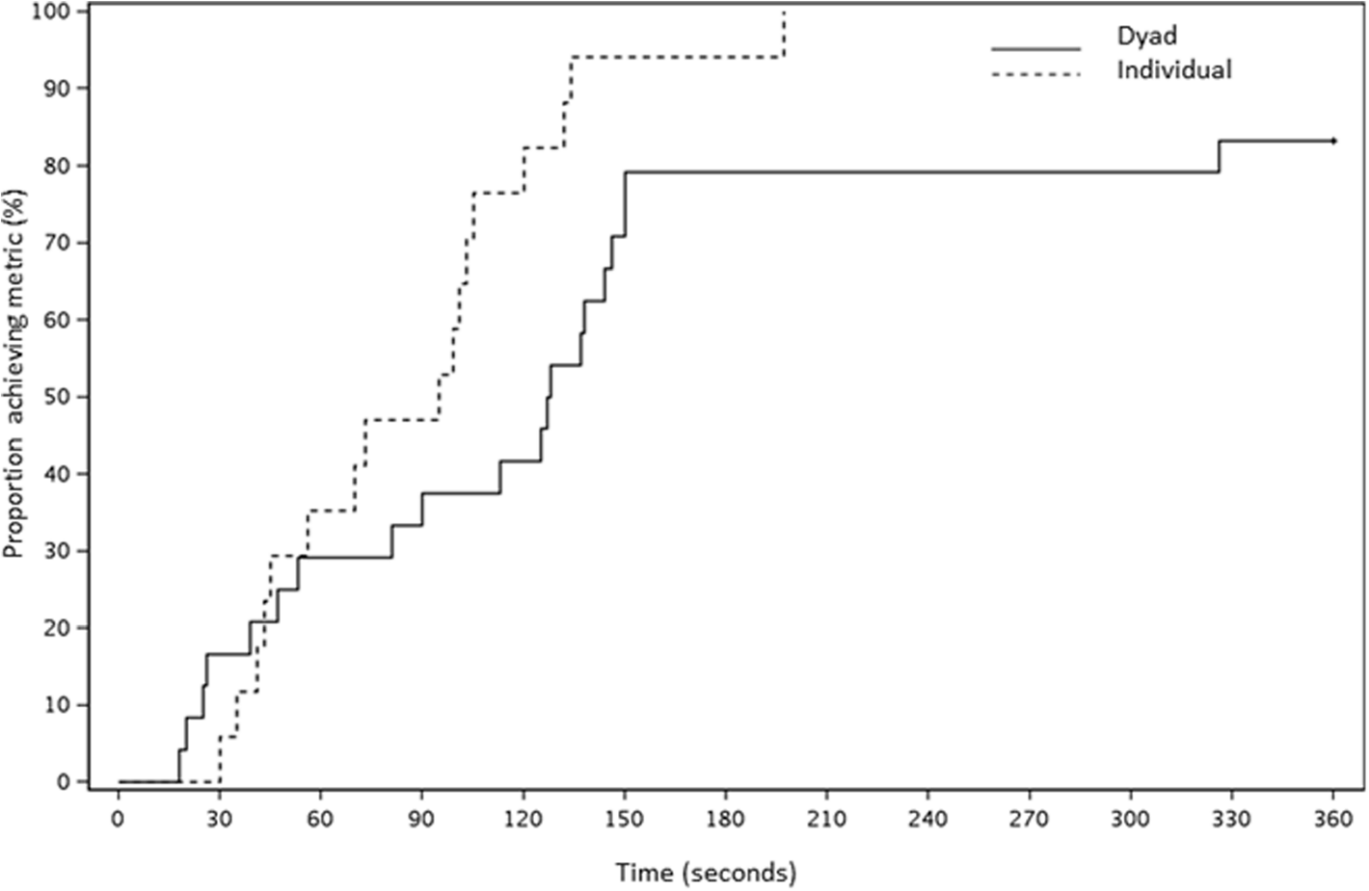
Kaplan-Meier curve of time (in seconds) to recognizing indication for transcutaneous pacing, Session #2, Dyads* vs Individuals (*Went through first session as a dyad. Session #2 performed as an individual)

The time to event analysis indicated differences on day 1 for the time to correct diagnosis of bradycardia, (hazard ratio [HR] = 3.32, *p* = 0.01) (Fig. 2) and time to recognizing TP indication (HR = 3.62, *p* = 0.04) (Fig. 3) between dyads and individuals, with results favoring dyads. There were also differences for time to effective TP (HR = 6.91), and time to calling for a rapid response or code team (HR = 1.70); however, these results were not statistically significant (*p* = 0.08 and 0.29, respectively). During the post-intervention assessment on day 4, time to recognizing the indication for TP was shorter for those in the individual training group (HR = 2.26, *p* = 0.02) (Fig. 4), but no other events had evidence of a difference (bradycardia HR = 1.2, *p* = 0.63; effective pacing HR = 1.5, *p* = 0.30; RRT HR = 1.03, *p* = 0.94).

While performance metrics were largely equivalent for all participants in individualized testing on day 4, there were significant differences in the degree of improvement between initial training as either dyads or as individuals and day 4 post-intervention testing. The proportional hazards model for the differential training effect between the randomized groups indicates the groups differed in their time to task completion for the time to correct diagnosis of bradycardia (*p* = 0.02), time to TP indication (*p* < 0.001), but in both cases the learning effect (day 1 versus day 4) has the greatest effect. For both diagnosis of bradycardia and time to TP indication, the individuals had a greater improvement in their time to task completion relative to dyads (bradycardia individual HR = 5.1 vs 3.1; pacing indication individual HR = 19.0 vs 8.1). Because those in the individual training group performed more poorly on initial testing, they had the most room for improvement.

Preference for dyad training was higher in those randomized to the dyad session (*p* = 0.03) with 58% of those in the dyad group preferring a paired learning session, whereas only 23% of those in the individual group reported they would have preferred training as a dyad (i.e., 77% of those randomized to an individual session reported a preference to continue training as an individual). Overall, 56% of students reported they would prefer to train as an individual.

## Discussion

The purpose of this study was to compare dyad and individual learning during a simulation-based bradycardia scenario to better understand the impact of dyad training on emotional state (stress, anxiety), cognitive load, and performance. This has not been previously examined in the literature. Prior studies have focused on the efficiency and efficacy of dyad training. As expected, on the initial day of training, students in the dyad groups had lower stress and anxiety levels and achieved better performance metrics when compared to individuals (i.e., teams of two individuals outperformed teams with only one individual). Interestingly, the cognitive load was identical in both groups. Stress, anxiety, and performance were similar on individual post-intervention testing on day 4.

We note that anxiety scores (per STAI) had a large standard deviation in both groups (± 15.8 for individuals, ± 14.4 for the dyads on an 80-point scale). We attribute this in part to the diversity of student personalities, prior clinical experiences, and widely varied preferences for specialty training. Studies conducted with more like participants, such as Harvey, et al. [15, 30] (which had only general surgery and emergency medicine residents), would likely not demonstrate such variability in subjective emotional state measures. The finding that students in the dyad groups had lower stress and anxiety levels for their initial training experience would support the theory that partnership reduces emotional turmoil in simulation scenario participants. In contrast to our hypothesis, this less stressful and less anxiety-provoking educational experience did not seem to translate to an overall lower performance on later individual testing. The single exception was time to recognizing the indication for TP, but students initially in a dyad otherwise had similar performance to individuals on post-intervention assessment on day 4. Prior studies have shown that moderately increased stress may improve memory consolidation [11, 13–15] which may suggest that the individuals should have performed better on post-intervention assessment on day 4 given their higher stress and anxiety scores on day 1. What is unclear, given the short duration of this study, is if this benefit would be borne out on retention testing done several weeks or months later, when a well-consolidated memory would be more important and the effect of stress on memory consolidation would be more obvious. Other areas of uncertainty are the motivation that each student had to study bradycardia or other ACLS algorithms after this scenario, which could have differed between the dyad and individual groups due to different levels of stress after day 1.

Studies have demonstrated that dyad training is an effective, or at least non-inferior, method of medical education in acquisition of procedural skills in bronchoscopy [6], coronary angiography [7], lumbar puncture [4], and ultrasound [8] as well as clinical encounter skills [5], with numerous theories for why this serves as an effective knowledge transfer. Due to the systematic nature of procedural skills training, it had been assumed that these scenarios were less stressful than the ACLS bradycardia scenario completed by our students, and thus these studies were not necessarily expected to relate to our more stressful scenario. However, there may have been more overlap in the emotional state involved in procedural training than expected, which may explain our results demonstrating an equivalent short-term performance between groups. Tolsgaard [8] raises the possibility that the time spent in dyad practice may be inversely related to the gains in learning of the dyad participants (i.e., shorter practice time yields greater benefit to dyads than longer sessions); our students’ scenario, at only 8 min actively engaged in simulation (plus an additional 8 min for the standardized video debrief), was the shortest of the known dyad studies, and thus would have expected greater gains by students in the dyad group, which was not borne out on our post-intervention testing. However, our study also was a less procedurally-focused topic than many of the above, raising some question of applicability of much of the prior literature. Cognitive load theory [18, 19, 36] suggests that a complex task like diagnosing and managing symptomatic bradycardia (particularly at the medical student level) may be well suited for learning as dyad. Tolsgaard [9] notes that dyad structure would provide a larger reservoir of cognitive capacity to utilize for information processing and may therefore improve learning. It was thus an unexpected finding that cognitive load was not statistically different between the dyad and individual groups on day 1 (or day 4). One would anticipate that cognitive load would be reduced in the dyad groups due to the collaborative experience and the ability to divide tasks. However, this assumption was not supported by our data. This may be due to the different type of task asked of these students (highly cognitive and minimally procedural, rather than mostly procedural), or could be related to our small sample size and risk for Type II error.

Interestingly, students reported a preference for working through this scenario as individuals (56%) instead of dyads, despite higher levels of stress and anxiety. Considering this was a low-stakes formative assessment, it may be that students liked the challenge of trying to perform alone and felt the increased stress more accurately represented real life scenarios they may face. Interpretation of this survey result is also challenging as students in the individual group did not get to experience a cross-over dyad session but were likely able to answer this question based on prior simulation based educational experiences both alone and with partners.

In the current climate of healthcare education, costs continue to climb while resources remain limited. If dyad training is as effective as individual training, this could be helpful in scheduling students more efficiently, as twice the number of learners would be able to utilize the same volume of resources. However, this study was not designed to investigate the learning effect, and we cannot conclude equivalency from our study, particularly due to our small sample size.

It remains to be determined if larger collections of learners (groups of 3 or more) could be taught simultaneously using SBME with similar educational outcomes or whether those simply observing a simulation scenario and participating in the debrief would have similar benefit. Other future directions for research include: comparison of training preference, emotional state, perceived cognitive load, and performance by future specialty; comparison of individuals, dyads, larger groups, and observers; and delayed retention testing. Further research is needed to clarify the optimal role of collaborative versus individual learning for non-procedural skills (as in this study) compared to more hands-on procedural skills.

### Limitations

The limitations of this study include its small sample size, relatively short time to post-intervention assessment, performance scoring by single, unblinded investigators who were also the course directors, the absence of specific instructions to collaborate during the scenarios for the dyad group, and the subjective survey method for measurement of workload, anxiety, and stress. We also used an abbreviated version of the STAI tool which could have an impact in anxiety measurement. While also validated tools, the Paas and CAR instruments contain few items within each measure (one and two questions, respectively) which may limit their effectiveness in this small sample size. Sample size was limited by class size, making it a fixed limitation and difficult to otherwise address in this single-site study; however, all the available students participated in this study. Adding additional class years in the future and expanding the course to other educational institutions would reduce the risk of Type II error and allow for further sub-analyses by gender or future specialty, for instance. The short time to post-intervention assessment was due to preset constraints of the IBC schedule. The medical school required that IBC be completed in the period of 1 week for each group of students. Moreover, IBC is held just prior to graduation, thus there was not a feasible opportunity to delay retention testing to a time outside of the week of the scheduled IBC. It is possible that there may be retention differences between those who went through the initial simulation as dyads and those who went through individually if we were able to assess performance at a more distant time. Additionally, determination of certain time metrics, such as time to awareness of bradycardia and time to recognizing an indication for TP, required verbalization from the participant or interpretation by the scorer, which may have led to unintentional confounding.

Finally, it is worth noting that the stress, anxiety, and cognitive load that a learner experiences while undergoing simulation-based education may not accurately reflect these measures if the learner were experiencing the same scenario in real life. The learning effect from the potentially different emotional state may cause either an improvement or detriment to memory consolidation [28–30]. It is also likely that a higher-level trainee, such as resident or fellow, would have a lower stress response to this complex patient scenario; if so, it is also unknown what impact this emotional state would have on learning.

## Conclusions

For our medical student cohort who underwent SBME of symptomatic bradycardia, dyad compared to individual training resulted in smaller changes in stress and anxiety from baseline while having minimal observed negative impact on clinically relevant objective performance measures. Dyad training appears to be an effective and efficient tool for SBME of symptomatic bradycardia for novice learners.

## Supplementary Information

**Additional file 1.**

## Data Availability

The datasets generated and/or analyzed during the current study are available from the corresponding author on reasonable request.

## Abbreviations

ACLS: Advanced Cardiac Life Support
CAR: Cognitive Appraisal Ratio
HR: Hazard ratio
HPE: Health professions education
IBC: Internship Boot Camp
IRB: Institutional Review Board
RRT: Rapid response team
SBME: Simulation based medical education
STAI: Abbreviated State-Trait Anxiety Inventory
TP: Transcutaneous pacing
