# Supplementary material for: Comparison of dyad versus individual simulation-based training on stress, anxiety, cognitive load, and performance: a randomized controlled trial
Source: BMC Med Educ. 2021 Jul 5;21:367. doi: 10.1186/s12909-021-02786-6 (PMC8256490; doi:10.1186/s12909-021-02786-6)
Supplement: Supplementary file 1 — Additional file 1. [file 12909_2021_2786_MOESM1_ESM.docx]

**APPENDIX**

1. **SESSION SCRIPT AND SCORING SHEET**

Student randomization ID number(s): **______________________ ______________________**

Opening vitals

| T | P | BP | RR | SaO2 | Wt |
| --- | --- | --- | --- | --- | --- |
| 36.8 | 30 | 88/55 | 18 | 98% | 80 kg |

Displayed rhythm: 3^rd^ degree AV block, rate 30

Session/timer begins when student enters room. Simulation tech playing an RN is in room, but answers no questions and gives only the prompts below when/if indicated (given by scorer via headset; see “allowed responses” below).

**AT TWO** MINUTES:

- *RN prompts if not yet identified: “His heart rate is pretty low”
- **Time** at diagnosis of **bradycardia**: _________________________ *(or note if not diagnosed by learner)*

**AT FOUR** MINUTES:

- *If student(s) not yet pacing, select “lower BP” phase: Eyes close, BP lowers to 74/40. HR same.

**AT SIX** MINUTES:

- *If not yet pacing, RN prompts: “Should we try using the pacemaker?” and points to pacer
- **Time** when acknowledge need **to use pacer**:______________ *(or note if not indicated by learner)*
- **Directs to place pads correctly Y/N**
- **Turns on pacer function Y/N**
- **Selects/leaves at appropriate rate Y/N [60-100]**
- **Selects appropriate current Y/N [80-90] Current selected ___________**
- **Time** at effective pacing (per facilitators): ________________ *(or note if no effective pacing)*
- **Identifies capture Y/N**
- Move to “higher BP” phase once effective pacing. Eyes open, BP improves to 98/60, heart rate as paced
- **Independently calls RRT /code Y/N. Time** **at RRT/code call**:______________________ *(or note if not called by learner)*
- **Considers analgesia for patient Y/N**
  - Prompt by having patient voice “ouch, that hurts” after capture going x 1 minute

**AT SEVEN** MINUTES:

- If code or RRT not called already, RN prompts: “I’m going to call a code to get more help.”

**AT EIGHT** MINUTES:

- Code team arrives, case ends (if objectives not met). Code team **asks “What was the rhythm?”** 🡪 **Correct Diagnosis Y/N**

ALLOWED PATIENT RESPONSES (Voiced by TL or EA):

- - **“I have been weak for the last 2 days, and it’s getting worse”**
  - If more details asked, will just say: **“I don’t know, I just feel so weak.”**
  - If intern continues to probe with history, will say: **“I feel like I am going to pass out.”**
  - If asking about medical history, meds, etc., just say: **“It’s in my chart.”**

1. **VIDEO DEBRIEF**

All students viewed the same video after their first session. There was no in-person debrief. The video covered the following:

1. Assessing airway, breathing, circulation
2. Determining whether bradycardia was symptomatic or asymptomatic
3. Reviewing ECG interpretation and differential diagnosis
4. Review of ACLS algorithm
   1. Pharmacologic management
   2. Types of pacing and how to apply
   3. How to use the Zoll pacer function
5. After care
6. **SURVEY INSTRUMENTS**

**Bootcamp Simulation Education Pre-Scenario Survey**

1a.- How stressful do you expect the upcoming task to be? (Please circle one option)

1 2 3 4 5 6

*Very low task-induced stress Very high task-induced stress*

1.b.- How able are you to cope with this upcoming task? (Please circle one option)

1 2 3 4 5 6

*Very incapable to perform the task Very capable to perform the task*

2.- Please complete the following 6 items according to how you currently feel (Please select one option per item):

a.- I feel calm

1 2 3 4

*not at all very much*

b.- I am tense

1 2 3 4

*not at all very much*

c.- I feel upset

1 2 3 4

*not at all very much*

d.- I am relaxed

1 2 3 4

*not at all very much*

e.- I feel content

1 2 3 4

*not at all very much*

f.- I am worried

1 2 3 4

*not at all very much*

**Bootcamp Simulation Education Post Scenario Survey**

1.- Please rate the perceived intensity of your mental effort during the simulated scenario (Please select one option):

€ Very, very low mental effort € Very low mental effort

€ Low mental effort € Rather low mental effort

€ Neither low nor high mental effort

€ Rather high mental effort

€ High mental effort

€ Very high mental effort

€ Very, very high mental effort

2a.- How stressful was the task performed? (Please circle one option)

1 2 3 4 5 6

*Very low task-induced stress Very high task-induced stress*

2.b.- How able were you to cope with the task performed? (Please circle one option)

1 2 3 4 5 6

*Very incapable to perform the task Very capable to perform the task*

3.- Please complete the following 6 items according to how you feel after the simulated scenario (Please select one option per item):

a.- I feel calm

1 2 3 4

*not at all very much*

b.- I am tense

1 2 3 4

*not at all very much*

c.- I feel upset

1 2 3 4

*not at all very much*

d.- I am relaxed

1 2 3 4

*not at all very much*

e.- I feel content

1 2 3 4

*not at all very much*

f.- I am worried

1 2 3 4

*not at all very much*

1. **SATISFACTION SURVEY**

I was given the necessary education and training to manage a patient with symptomatic bradycardia

| Strongly Disagree | Disagree | Undecided | Agree | Strongly Agree |
| --- | --- | --- | --- | --- |
| 1 | 2 | 3 | 4 | 5 |

How would you rate the training received to manage a patient with symptomatic bradycardia?

| Very poor | Poor | Undecided | Good | Very good |
| --- | --- | --- | --- | --- |
| 1 | 2 | 3 | 4 | 5 |

Overall, how would you rate your experience with the education process related to managing a patient with symptomatic bradycardia?

| Very poor | Poor | Undecided | Good | Very good |
| --- | --- | --- | --- | --- |
| 1 | 2 | 3 | 4 | 5 |

Would you recommend the education process related to managing a patient with symptomatic bradycardia to another person?

| Strongly Disagree | Disagree | Undecided | Agree | Strongly Agree |
| --- | --- | --- | --- | --- |
| 1 | 2 | 3 | 4 | 5 |

How would you have preferred training during the simulated symptomatic bradycardia scenario on the first day? (Please select one option)

a. Individually: ____

b. With a partner: ____

Do you have any comments, suggestions or feedback related to your education and training on how to manage a patient with symptomatic bradycardia?

_____________________________________________________________________________________________________________________________________________________________________________________________________________________________________________________________________________________________________________________________________________________________________________________________________________________________________________________________________________________________________________________________________________________________________________________________________________________________________________________________________________________________________________________________________________________________________________________________
